# Supplementary material for: Quantitative and Predictive Modelling of Lipid Oxidation in Mayonnaise
Source: Antioxidants (Basel). 2021 Feb 15;10(2):287. doi: 10.3390/antiox10020287 (PMC7919052; doi:10.3390/antiox10020287)

Supplementary information

Table S1. Schematic overview of the different mayonnaise formulation sets used in this study.

Ingredient	Both LOOH >40 mmol/kg data and aldehyde data beyond onset for prediction model selection (3.3.)			LOOH data only to used for model selection (3.2.)
	set A (5 form.)	set B (7 form.)	set C (11 form.)	set D (234 form.)
	%w/w			
Rapeseed oil	65.0	75.0	78.0	65-78
Water	22.1	12.0	14.8	12-22
Sucrose	1.3	1.3	-	0-1.3
NaCl	1.8	1.8	1.8	1.8
Vinegar spirit 12%	2.7	2.6	0.7	0.7-2.6
Egg blend (whole egg + egg yolk)	6.7	6.7	-	0-6.7
Egg yolk	-	-	4.6	0-4.6
$CaNa_2EDTA$	0-0.0075	0-0.0075	0-0.0075	0-0.0075
Other chemical antioxidants (BHT, BHT, chlorogenic acid)	0	0	0	0-0.02
Natural antioxidants (plant extracts)	0-5	0-5	0	0-5

Table S2. Overview of the boundaries using for the fitting of both Gompertz (G) and Foubert (F) functions.

	**Lower limit**	**Upper limit**
A (both G and F)	50	70
K (both G and F)	0	0.5
T0 (G)	0	5
f0 (F)	0	2
n (F)	0	75

Table S3. BIC on the F4 model with different n-values. The displayed number represents the number of curves that have that model as the lowest criterion-value.

	n=8	n=16.7	n=25.8	n=36
BIC	45	109	31	56

Figure S1. Schematic overview on the cross-point determination. On the left, the squares represent the concentration of aldehydes measured over time. The dashed line the reduced Foubert (n=1.1) fit. The green line is the baseline, set at Cald = 1. The orange line is the result of the maximum slope determination using Eq.4, as shown in the right graph. The onset was determined by crossing the orange and green line.

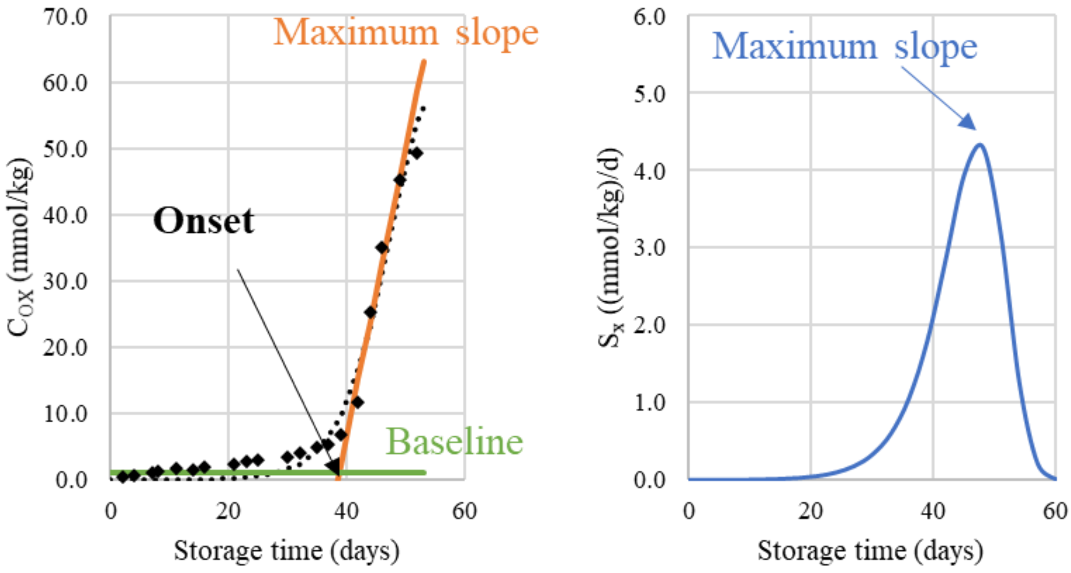

Figure S2. Statistical justification for the aldehyde onset prediction model. To verify whether it was justified to fit the linear regression of the prediction model through the origin, linear regression models with and without forcing the regression through the origin were compared to each other (Figure S5A/S5B). Both models fitted the data well, with comparable residual standard errors. No significant difference was found between the models by ANOVA analysis (Figure S7C). The residuals vs fitted plot (Figure S5D) showed a small trend, which did not worry us as we anticipated this due to using different formulations. The normal Q-Q plot (Figure S5E) showed that our data was normally distributed enough for this linear regression model.

A

```
> model.pred1 <- lm(Ald.onset ~ LOOH38, data = dat2)
> summary(model.pred2)

Call:
lm(formula = Ald.onset ~ LOOH38, data = dat2)

Residuals:
   Min     1Q Median     3Q    Max 
-4.243 -2.621 -1.195  2.929  5.714 

Coefficients:
            Estimate Std. Error t value Pr(>|t|)    
(Intercept)  1.64089    1.33516   1.229    0.233    
LOOH38       1.51765    0.08951  16.955 9.91e-14 ***
---
Signif. codes:  0 ‘***’ 0.001 ‘**’ 0.01 ‘*’ 0.05 ‘.’ 0.1 ‘ ’ 1

Residual standard error: 3.35 on 21 degrees of freedom
Multiple R-squared:  0.9319,	Adjusted R-squared:  0.9287 
F-statistic: 287.5 on 1 and 21 DF,  p-value: 9.906e-14
```

B

```
> model.pred2 <- lm(Ald.onset ~ 0 + LOOH38, data = dat2)
> summary(model.pred)

Call:
lm(formula = Ald.onset ~ 0 + LOOH38, data = dat2)

Residuals:
   Min     1Q Median     3Q    Max 
-3.765 -2.330 -0.586  3.215  6.587 

Coefficients:
       Estimate Std. Error t value Pr(>|t|)    
LOOH38  1.61140    0.04736   34.02   <2e-16 ***
---
Signif. codes:  0 ‘***’ 0.001 ‘**’ 0.01 ‘*’ 0.05 ‘.’ 0.1 ‘ ’ 1

Residual standard error: 3.388 on 22 degrees of freedom
Multiple R-squared:  0.9813,	Adjusted R-squared:  0.9805 
F-statistic:  1158 on 1 and 22 DF,  p-value: < 2.2e-16
```

C

```
> anova (model.pred1, model.pred2)
Analysis of Variance Table

Model 1: Ald.onset ~ LOOH38
Model 2: Ald.onset ~ 0 + LOOH38
  Res.Df    RSS Df Sum of Sq      F Pr(>F)
1     21 235.61                            
2     22 252.56 -1   -16.946 1.5104 0.2327
```

D

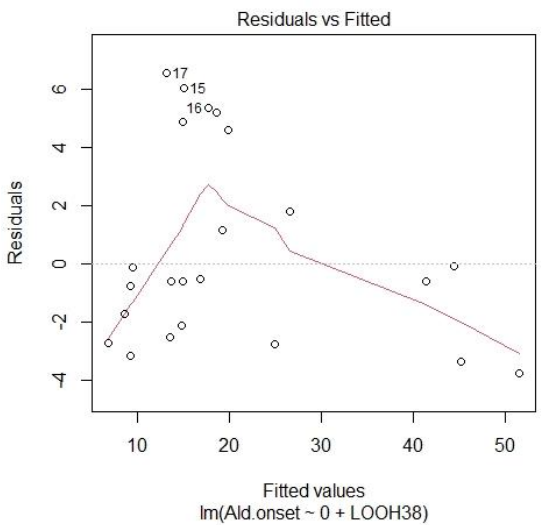

E

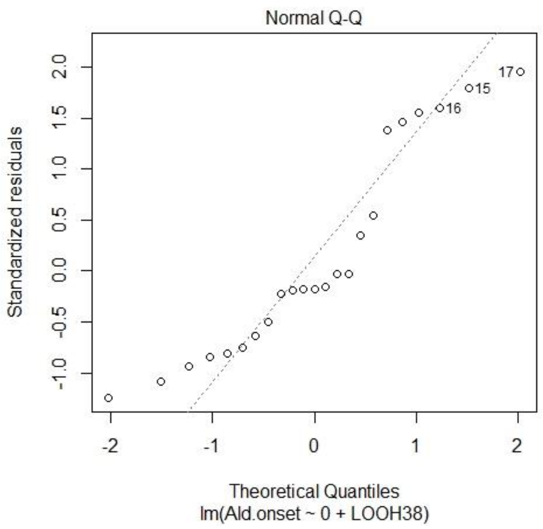

Figure S3. Reduced Foubert (F3, n = 16.7) regression results on the Hydroperoxide data by ^{1}H NMR up to 40 mmol/kg. The dots are the actual measured data points, the solid line the F3 function and the dashed lines represent the 95% CI.

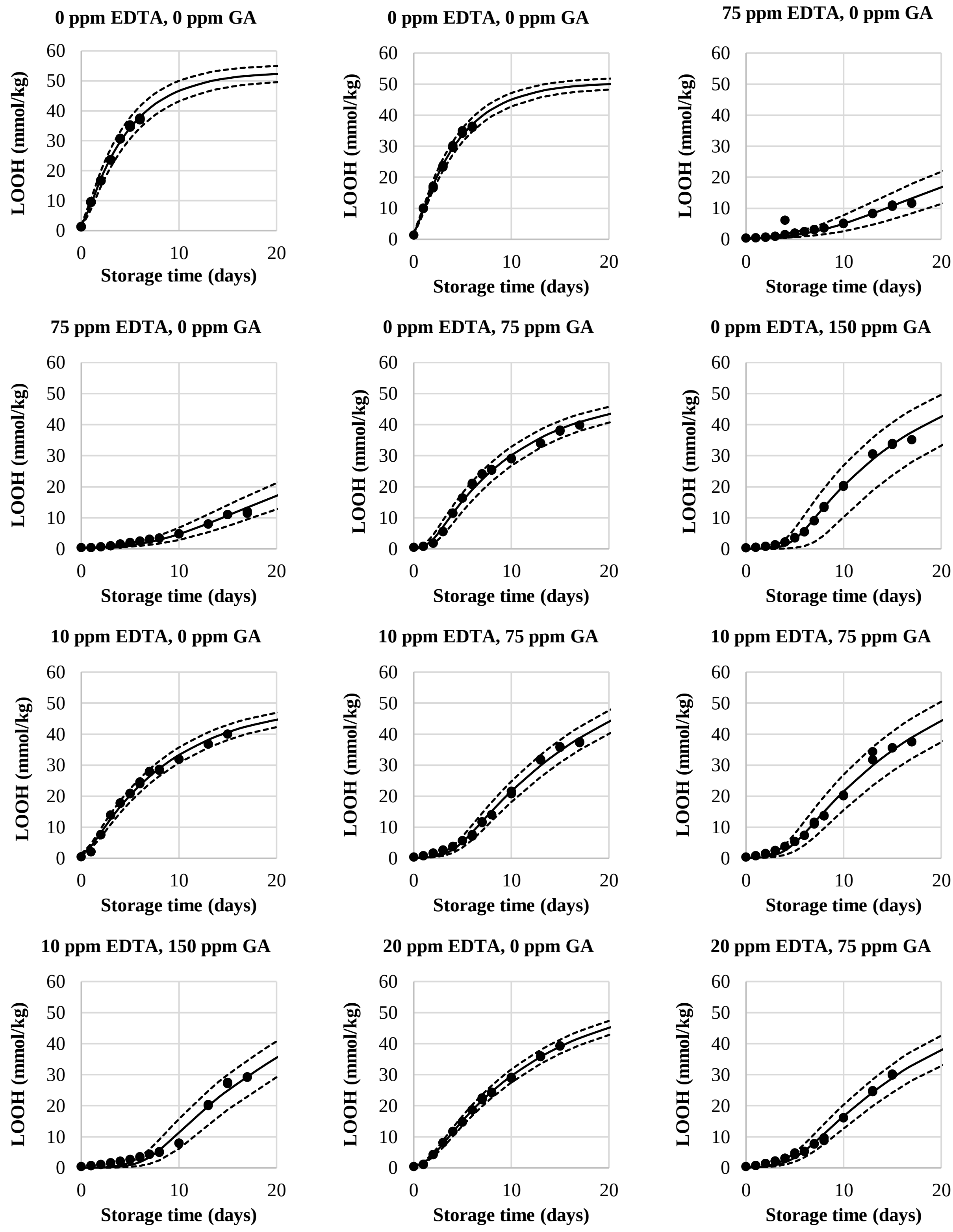

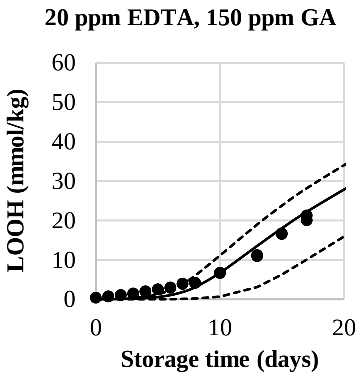

20 ppm EDTA, 150 ppm GA
LOOH (mmol/kg)
60
50
40
30
20
10
0
0
10
20
Storage time (days)

Figure S4. Reduced Foubert (F3, n = 1.1) regression results on the Aldehyde data by ^{1}H NMR up to 20 mmol/kg. The dots are the actual measured data points, the solid line the F3 function and the dashed lines represent the 95% CI.

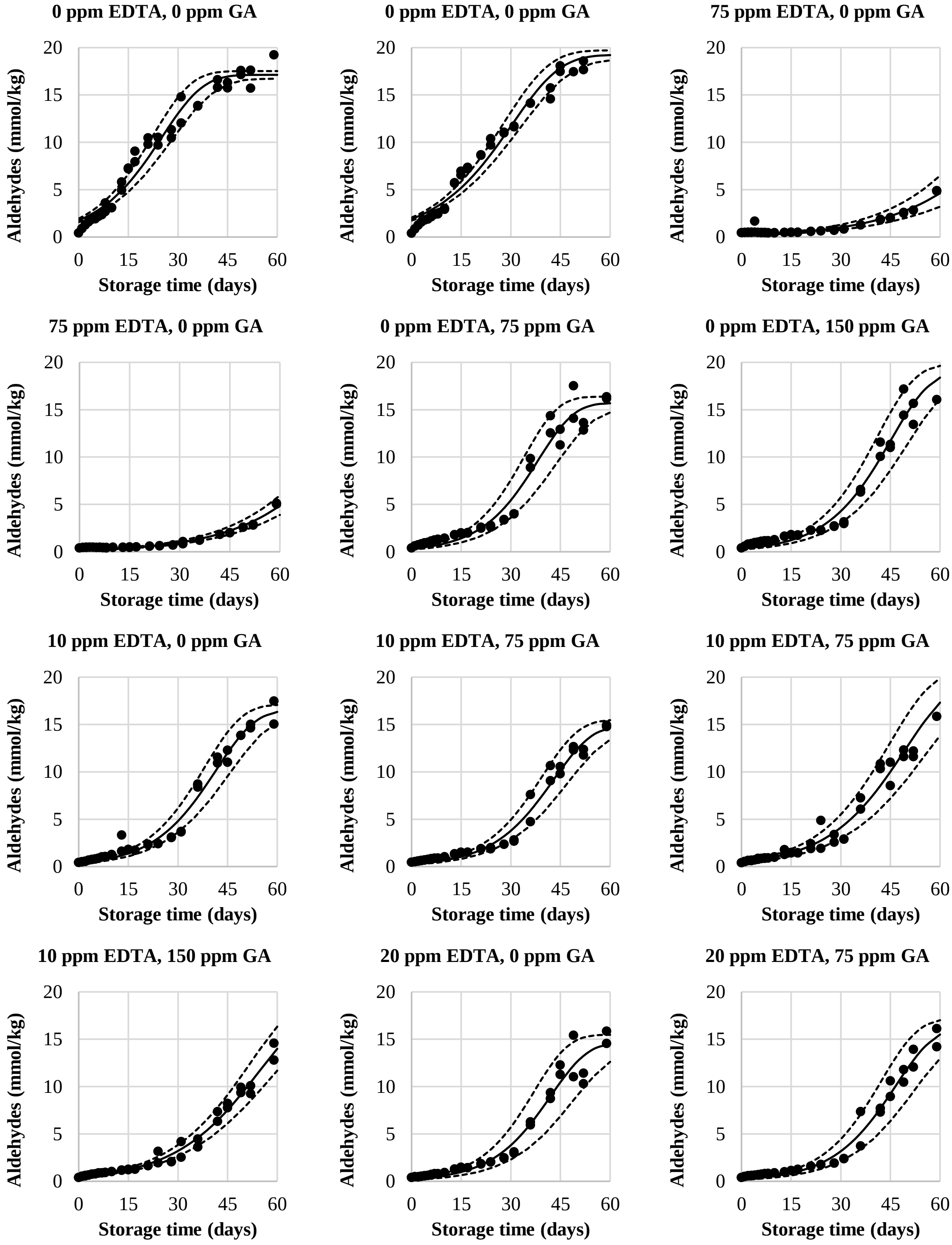

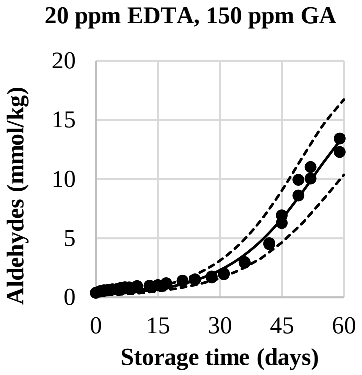
20 ppm EDTA, 150 ppm GA
Aldehydes (mmol/kg)
20
15
10
5
0
0
15
30
45
60
Storage time (days)

Figure S5. Overview of the parameters obtained from regression using the reduced Foubert (F3) function for the 'model validation' data set. A: f0, B: K. Shown error bars represent the 95% CI.

A

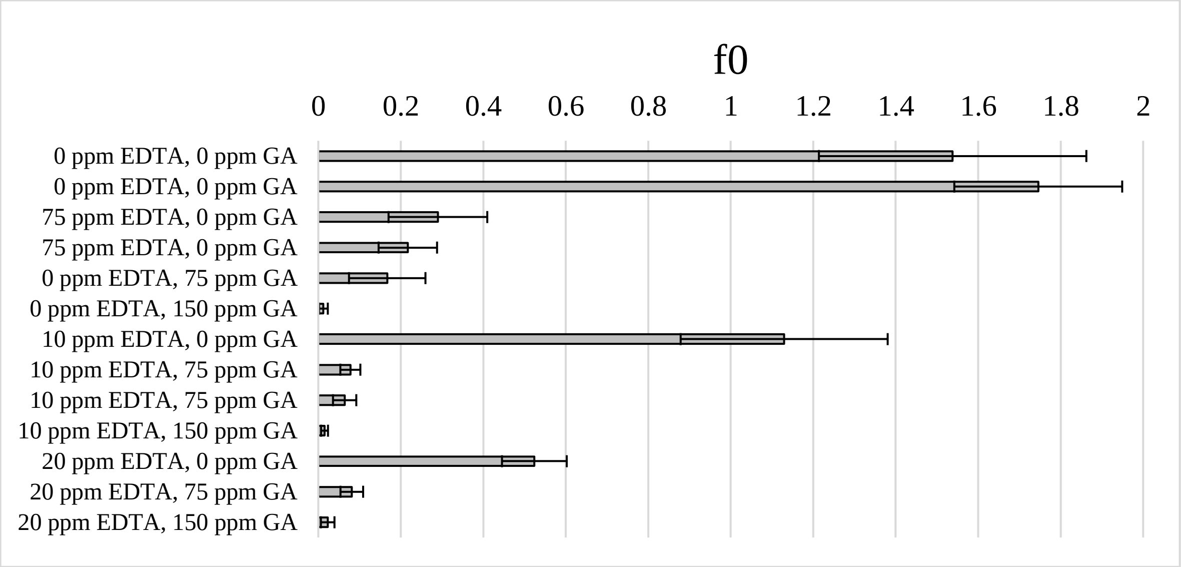

B

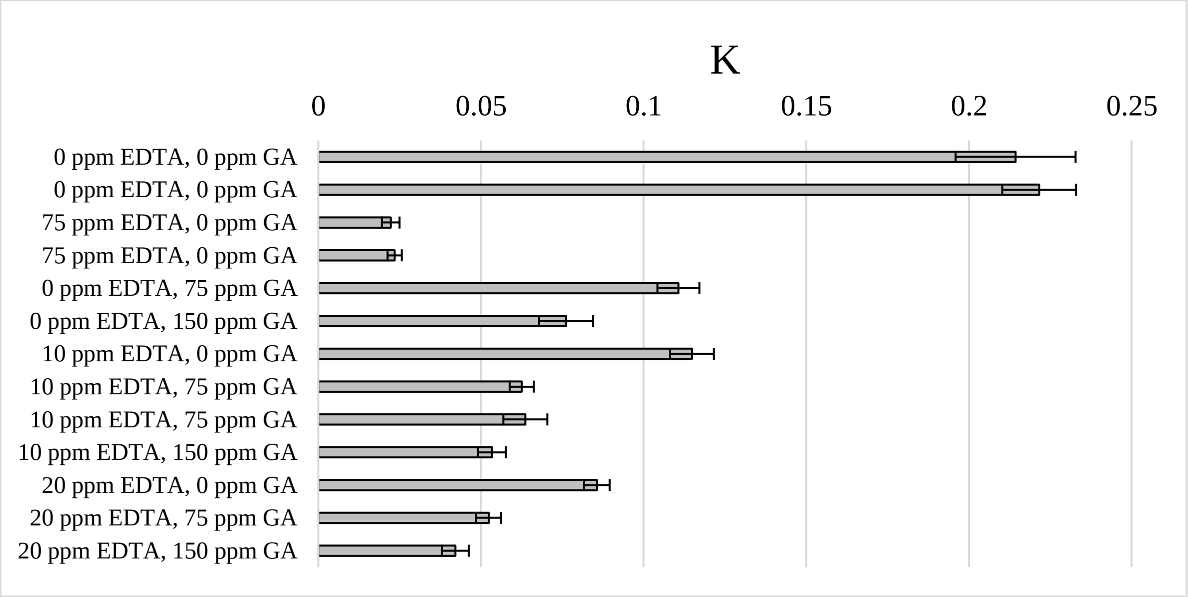

Supplement: Supplementary file 1 [file antioxidants-10-00287-s001.pdf]
